# Docking Analysis of Drugs Used in the Treatment of Alzheimer’s Disease, Using Cell Membrane Receptors and Enzymes of the Kynurenine Pathway: A Pilot Study

**DOI:** 10.3390/ijms27156555

**Published:** 2026-07-23

**Authors:** Abdulla A. B. Badawy, Shazia Dawood, Felix I. L. Clanchy, Richard O. Williams, Trevor W. Stone

**Affiliations:** 1 School of Health Sciences, Cardiff Metropolitan University, Western Avenue, Cardiff CF5 2YB, UK; 2 Pharmacy and Allied Health Sciences, Iqra University, Karachi 7580, Pakistan; shazia.dawood@iqra.ed.pk; 3 The Kennedy Institute of Rheumatology, University of Oxford, Oxford OX3 7FY, UK; felix.clanchy@kennedy.ox.ac.uk (F.I.L.C.); richard.williams@kennedy.ox.ac.uk (R.O.W.)

**Keywords:** acetylcholinesterase, Alzheimer’s disease, indoleamine 2,3-dioxygenase, docking, NMDA receptors, kynurenine, tryptophan 2,3-dioxygenase

## Abstract

Molecular docking in silico techniques were used to assess the interactions between several drugs used in the treatment of Alzheimer’s disease (AlzD) with neuronal receptors and with tryptophan and glycolytic pathway enzymes. AlzD drugs believed to act by inhibiting acetylcholinesterase (AChE) docked to that enzyme, whereas other drugs did not. No docking was observed to the nicotinic acetylcholine receptor (α-7-nAChR). All the drugs tested docked to the kainic acid receptor for glutamate, whereas donepezil, galantamine, tropisetron and N-acetylcysteine docked to the glutamate-NMDA receptor, but none docked to the glutamate-AMPA receptor. Two compounds docked to GABA receptors. Since the kynurenine pathway of tryptophan metabolism has been linked to AlzD, docking was examined with its various enzymes. Docking to tryptophan-2,3-dioxygenase (TDO2), indoleamine-2,3-dioxygenase-1 (IDO1) and 2-amino-3-carboxymuconic acid-6-semialdehyde decarboxylase was seen with most of the AlzD drugs. Donepezil docked to kynurenine monooxygenase, while rivastigmine, memantine and tropisetron docked to kynurenine aminotransferase-2. There was limited docking to the aryl hydrocarbon receptor and glycolytic enzymes, whereas most drugs docked to phosphoenolpyruvate carboxykinase (PEPCK). Despite their strong docking ability to IDO1 and TDO, none of the compounds tested inhibited their enzyme activity in an assay of kynurenine production. This pilot study highlights the varied pharmacological profile of drugs used in AlzD and suggests that a detailed examination of their functional effects should be considered to assist understanding of their different profiles at on- and off- target sites in human treatment.

## 1. Introduction

Cognitive impairment is a key feature of Alzheimer’s disease (AlzD) and related dementias [1,2]. The role of acetylcholine in memory has led to the development of therapeutic agents that inhibit its degradation by acetylcholinesterase (AChE) or activate its synaptic nicotinic (nAChR) or muscarinic receptors [3]. These sites affect neuronal excitability and plasticity, often by modulating glutamate release [4,5]. Several of these compounds are in clinical use, including donepezil [6], rivastigmine [7], varenicline [8] and tropisetron [9]. Galantamine is often used for its ability to inhibit AChE and to potentiate nAChR activity, although the latter claim has been disputed [10]. Memantine, which is primarily an antagonist at N-methyl-D-aspartate (NMDA) receptors, is also in clinical use, often in conjunction with an AChE inhibitor [11], while agonists at AMPA-sensitive glutamate receptors (‘ampakines’) are also of potential value [12].

Amyloid-β (Aβ), which features prominently in AlzD pathophysiology, suppresses synaptic activity partly by interacting with the α-7nAChR and glutamate-NMDA receptors [13]. Dissociating Aβ from these receptors partially normalizes their function. Upregulation of α-7-nAChR in astrocytes correlates with Aβ pathology [14], while antibodies to Aβ such as aducanumab [15] and lecanemab [16], have modest activity in early AlzD and have been clinically approved in some countries.

Other neuroactive compounds implicated in AlzD include γ-aminobutyric acid (GABA), with down-regulation of GABA transaminase, loss of GABAergic neurons and altered GABA receptor function [17]. Serotonin (5-hydroxytryptamine, 5-HT) may also be involved since low levels of 5-HT and its membrane transporters have been reported in AlzD, with a loss of 5-HT-releasing neurons [1,17].

One family of compounds of increasing interest in the understanding and treatment of cognitive disorders, neurodegeneration and associated disorders is the products of tryptophan metabolism along the kynurenine pathway [18,19]. Among the most prominent and intensively studied are quinolinic acid and kynurenic acid. The former is a selective endogenous agonist at the NMDA-sensitive glutamate receptors, while kynurenic acid is an antagonist at NMDA and AMPA receptors [20], with additional effects on the Aryl Hydrocarbon Receptor (AHR) [21] and the G-protein coupled site GPR35 [22]. The fundamental functional importance of the kynurenine pathway has been advocated in the concept of an organism-wide stress reflex compensation system, with kynurenic acid and quinolinic acid as major modulators of excitability and plasticity [23]. A particular relevance to Alzheimer’s disease is that quinolinic acid accumulation may contribute to neuronal damage and degeneration in the later stages of the disease, especially if there is an overall increase in the ratio of quinolinic acid to the neuroprotective antagonism by kynurenic acid.

The involvement of multiple neurotransmitters in AlzD pathophysiology probably accounts for the limited therapeutic response usually associated with single medication treatment, necessitating the use of combination therapy [24]. Galantamine plus memantine, for example, have been used in patients with AlzD [25,26,27] and other cognitive disorders with encouraging results [28] while combining donepezil with memantine has proved valuable for moderate to severe AlzD [29]. Various combinations of other drugs have been suggested for use in mild cognitive impairment [24].

The choice of appropriate and effective combinations is hampered by the limited information on drug actions other than those on AChE and its receptors and by the potential interactions between neurotransmitter systems, such as those for acetylcholine and glutamate [4,5]. The aim of the present study is to perform an initial examination of the ability of some of the drugs already in use for AlzD to bind to a range of molecular targets, including the main enzymes in the kynurenine pathway. This preliminary study has been limited to an examination of the binding energy of compounds using in silico receptor docking technology, with an additional examination of enzyme activity of the key enzymes in tryptophan metabolism, indolamine-2,3-dioxygenase-1 (IDO1) and tryptophan-2,3-dioxygenase (TDO2).

## 2. Results

Docking of the six drugs used in the treatment of Alzheimer’s disease and of the proposed adjunctive antioxidant N-acetylcysteine (NAC) is summarized in Table 1, with further details on each of the compounds in Table 2, Table 3, Table 4, Table 5, Table 6, Table 7 and Table 8.

**Table 1:** Molecular docking of drugs to the crystal structures of enzymes and receptors. Data are shown as re-ranked docking scores (Kcal/mol). Docking score is inversely proportional to the degree of binding, with more negative values indicating stronger docking. For abbreviations, see Table 9.

	Re-Ranked Docking Scores (Kcal/mol)						
Drug	Donepezil	Galantamine	Rivastigmine	Memantine	Tropisetron	Varenicline	NAC
Enzyme							
AChE	−106.5	−23.6	−102.4	-	-	-	-
TDO2	-	−58.0	−73.3	−61.9	−79.3	−76.7	−70.4
IDO1	−108.7	−89.2	−84.7	−63.8	−99.2	-	-
KMO	−55.9	−87.8	-	-	-	-	-
KAT-2	−98.5	-	−70.0	-	−70.2	−58.5	−52.4
KYNU	-	-	-	-	-	-	-
3-HAAO	-	-	-	-	-	-	-
ACMSD	−105.9	-	-	-	−100.9	−74.3	−53.7
QPRT	-	−83.8	−68.5	-	−21.2	-	−61.9
PPAR-α	-	-	-	-	-	-	
G-6-Pase	-	-	-	-	-	-	−59.6
Hexokinase	-	-	-	-	-	-	-
LDH	-	−76.1	-	-	-	-	-
PEPCK	−92.4	-	−75.2	−46.4	−69.3	−31.1	−55.4
**Receptor**							
AHR	-	−89.3	−78.6	-	-	-	-
NMDA	−86.9	−89.3	-	-	−93.2	-	-
Kainate	−50.9	−60.7	−83.6	−59.3	−42.8	−55.1	−61.7
AMPA	-	-	-	-	-	-	-
GABA_A_	−106.8	-	-	-	−80.7	-	-
GABA_B_	-	-	-	-	−72.1	−71.6	-
α7-nAChR	-	-	-	-	-	-	-

**Table 2:** Docking parameters of donepezil to AChE and other enzymes and neuronal receptors (Kcal/mol). Docking score is inversely proportional to the degree of binding, with more negative values indicating stronger docking. RMSD (root mean square deviation). For abbreviations, see Table 9.

Enzyme	Docking Score	Re-Rank Score	RMSD	Torsion Energy	H Bond
AChE	−128.1	−106.5	0	6	−1.7403
IDO1	−140.0	−108.7	0	6	−2.122
KMO	−136.4	−55.9	0	6	−2.249
KAT-2	−125.7	−98.5	0	6	−1.341
ACMSD	−131.1	−105.9	0	6	0
PEPCK	−119.8	−92.4	0	6	−0.425
**Receptor**					
NMDA	−131.3	−86.9	0	6	−2.489
kainate	−125.0	−50.9	0	6	−1.683
GABA_A_	−138.4	−106.8	0	6	−0.764

**Table 3:** Docking parameters of galantamine to AChE and other enzymes and neuronal receptors (Kcal/mol). Docking score is inversely proportional to the degree of binding, with more negative values indicating stronger docking. RMSD (root mean square deviation). For abbreviations, see Table 9.

Enzyme	Docking Score	Re-Rank Score	RMSD	Torsion Energy	H Bond
AChE	−111.4	−23.6	0	1	−0.883
TDO	−127.1	−58.0	0	1	−2.893
IDO1	−120.6	−89.2	0	1	−2.392
KMO	−108.5	−87.8	0	1	−3.156
QPRT	−110.2	−83.8	0	6	−5.039
AHR	−108.0	−89.3	0	1	−2.323
LDH	−97.9	−76.1	0	1	−2.500
**Receptor**					
NMDA	−111.3	−89.3	0	1	−2.801
kainate	−124.9	−60.7	0	1	−8.176

**Table 4:** Docking parameters of rivastigmine to AChE, kynurenine pathway and other enzymes and neuronal receptors (Kcal/mol). Docking score is inversely proportional to the degree of binding, with more negative values indicating stronger docking. RMSD (root mean square deviation). For abbreviations, see Table 9.

Enzyme	Docking Score	Re-Rank Score	RMSD	Torsion Energy	H Bond
AChE	−121.0	−102.4	0	5	−0.97
TDO	−113.5	−73.3	0	5	0
IDO1	−113.2	−84.8	0	5	0
KAT-2	−85.4	−70.0	0	5	−0.306
QPRT	−91.8	−68.5	0	5	−1.52
AHR	−91.3	−78.6	0	5	0
PEPCK	−86.1	−75.2	0	5	−4.394
**Receptor**					
kainate	−106.3	−83.6	0	5	−5.757

**Table 5:** Docking parameters of memantine to AChE, kynurenine pathway and other enzymes and neuronal receptors (Kcal/mol). Docking score is inversely proportional to the degree of binding, with more negative values indicating stronger docking. RMSD (root mean square deviation). For abbreviations, see Table 9.

Enzyme	Docking Score	Re-Rank Score	RMSD	Torsion Energy	H Bond
TDO	−85.5	−61.9	0	0	−1.169
IDO1	−87.5	−63.8	0	0	−2.50
PEPCK	−47.2	−46.4	0	0	−2.716
**Receptor**					
Kainate	−75.0	−59.3	0	0	−4.218

**Table 6:** Docking of tropisetron to the kynurenine pathway and other enzymes and neuronal receptors (Kcal/mol). RMSD (root mean square deviation). Docking score is inversely proportional to the degree of binding, with more negative values indicating stronger docking. For abbreviations, see Table 9.

Enzyme	Docking Score	Re-Rank Score	RMSD	Torsion Energy	H Bond
TDO	−150.0	−79.3	0	3	−2.70
IDO1	−131.8	−99.2	0	3	−0.369
KAT-2	−102.0	−70.2	0	3	−1.773
ACMSD	−122.6	−100.9	0	3	−0.836
QPRT	−105.8	−21.2	0	3	−3.192
PEPCK	−103.1	−69.3	0	3	−4.039
**Receptor**					
NMDA	−121.0	−93.2	0	3	0
Kainate	−116.1	−42.8	0	3	−7.292
GABA_A_	−100.9	−80.7	0	3	−4.327
GABA_B_	−120.3	−72.1	0	3	−2.975

**Table 7:** Docking of varenicline to AChE and other enzymes and neuronal receptors (Kcal/mol). RMSD (root mean square deviation). Docking score is inversely proportional to the degree of binding, with more negative values indicating stronger docking. For abbreviations, see Table 9.

Enzyme	Docking Score	Re-Rank Score	RMSD	Torsion Energy	H Bond
TDO	−107.2	−76.7	0	0	−2.521
KAT-2	−73.1	−58.5	0	0	−3.372
ACMSD	−82.0	−74.3	0	0	−2.401
PEPCK	−83.6	−31.1	0	0	−4.925
**Receptor**					
Kainate	−83.8	−55.1	0	0	−4.156
GABA_B_	−85.8	−71.6	0	0	−4.526

**Table 8:** Docking of N-acetylcysteine (NAC) to AChE, kynurenine pathway and other enzymes and neuronal receptors (Kcal/mol). RMSD (root mean square deviation). Docking score is inversely proportional to the degree of binding, with more negative values indicating stronger docking. For abbreviations, see Table 9.

Enzyme	Docking Score	Re-Rank Score	RMSD	Torsion Energy	H Bond
TDO	−93.3	−70.4	0	3	−6.616
KAT-2	−64.2	−52.4	0	3	−5.972
ACMSD	−71.8	−53.7	0	3	−2.465
QPRT	−70.3	−61.9	0	3	−7.175
PEPCK	−72.2	−55.4	0	3	−9.535
G-6-Pase	−74.1	−59.6	0	3	−5.366
**Receptor**					
Kainate	−80.8	−61.7	0	3	−12.670

**Table 9:** Sources of enzymes and receptors, their PDB ID numbers, and references. The abbreviation and name of the enzymes and receptors employed, including species of origin and their PUBMED ID numbers.

Abbrev.	Enzyme and Receptor	Source	PDB ID	Reference
TDO2	Tryptophan 2,3-dioxygenase	*Xanthomonas campestris*	2NW8	[30]
IDO1	Indoleamine 2,3-dioxygenase	*Homo sapiens*	2D0T	[31]
KMO	Kynurenine 3-monooxygenase	*Pseudomonas fluorescens*	5X6Q	[32]
AADAT	Kynurenine aminotransferase 2	*Homo sapiens*	5EUN	[33]
KYNU	Kynureninase	*Homo sapiens*	2HZP	[34]
3-HAAO	3-Hydroxyanthranilate-3,4-dioxygenase	*Homo sapiens*	2qnk	[35]
ACMSD	Aminocarboxymuconate-semialdehyde decarboxylase	*Homo sapiens*	7PWY	[36]
QPRT	Quinolinate phosphoribosyltransferase	*Homo sapiens*	4KWW	[37]
ACHE	Acetylcholine esterase	*Homo sapiens*	4M0E	[38]
AHR/RR	Aryl hydrocarbon receptor: AHR/ARNT	*Homo sapiens*/*Bos taurus*	5Y7Y	[39]
PPARA	Peroxisome proliferator-activated receptor-α	Synthetic derivative	7E5H	[40]
G-6-Pase	Glucose-6-phosphatase	*Homo sapiens*	AF_AFQ9NQR9F	[41]
Hex K2	Hexokinase II	*Homo sapiens*	5HEX	[42]
LDHA	Lactate dehydrogenase A	*Homo sapiens*	4OJN	[43]
PEPCK	Phosphoenolpyruvate carboxy kinase	*Escherichia coli* K-12	1AQ2	[44]
GluN2B	N-methyl D-aspartate receptor subtype 2B	*Rattus norvegicus*	3JPW	[45]
GABRB3	GABA(A)R-beta3 homopentamer	*Homo sapiens*	4COF	[46]
GBR2	GABA(B) receptor	*Homo sapiens*	4F11	[47]
GRIK2	Glutamate ionotropic receptor kainate subunit 2	*Rattus norvegicus*	8FWR	[48]
GLUA2	Glutamate ionotropic receptor AMPA subunit 2	*Rattus norvegicus*	5VHZ	[49]
CHRNA7	Neuronal acetylcholine receptor subunit alpha-7	*Homo sapiens*	7KOX	[50]

### 2.1. Docking to Acetylcholinesterase (AChE)

**Figure 1:**
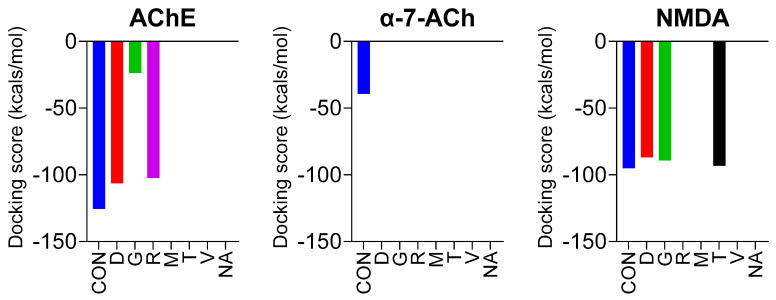

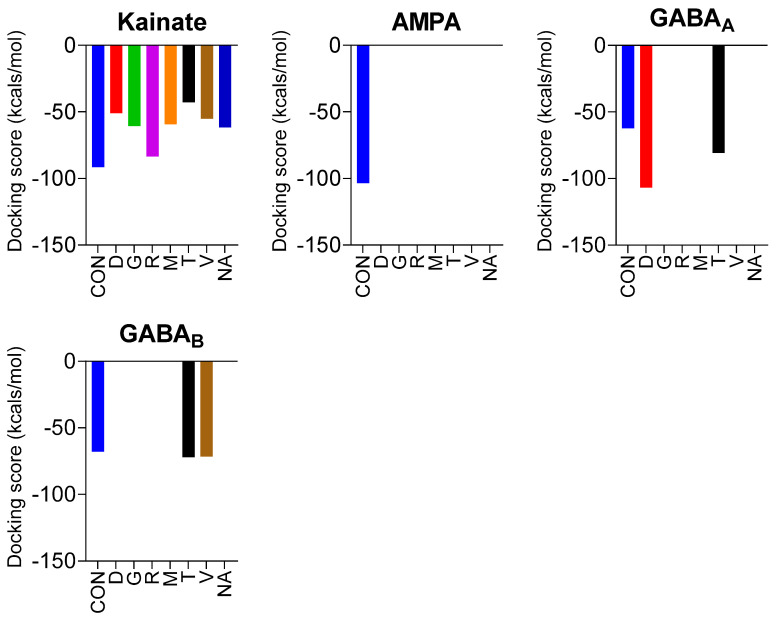
AlzD drugs dock to acetylcholinesterase and neuronal receptors. Docking scores for the binding of AD drugs to acetylcholinesterase (AChE) and neuronal receptors. Docking score is inversely proportional to the degree of binding, with more negative values indicating stronger docking. Drugs are indicated as follows: D—donepezil; G—galantamine; R—rivastigmine; M—memantine; T—tropisetron; V—varenicline; NA—N-acetylcysteine. CON refers to the routine control ligand for each target, listed in Supplementary Table S1.

Donepezil did not dock at the AChE active site (Figure 1, Table 2), but docked at an allosteric site with a different set of amino acid residues (Arg296, Pro368, His405, Trp532, Val370, Pro235, Asn533, Leu536) that characterize one of several allosteric sites [51]. Only two of the established AChE inhibitors—galantamine (Table 3) and rivastigmine (Table 4)—docked to AChE, with galantamine exhibiting a weaker (less negative) docking score. The remaining four drugs did not dock (Figure 1).

### 2.2. Docking to α-7-nAChRs and Glutamate NMDA Receptors

With NMDA receptors, docking was observed with all compounds except rivastigmine, memantine (Table 5) and varenicline (Figure 1). The strongest docking to NMDA receptors was with tropisetron (Table 6), and the weakest was with N-acetylcysteine. No docking to α-7-nAChR was observed with any of the seven compounds (Table 2, Figure 1), although tropisetron (Table 6) and varenicline (Table 7) are known to be agonists at that receptor.

### 2.3. Docking to Other Receptors

**Figure 2:**
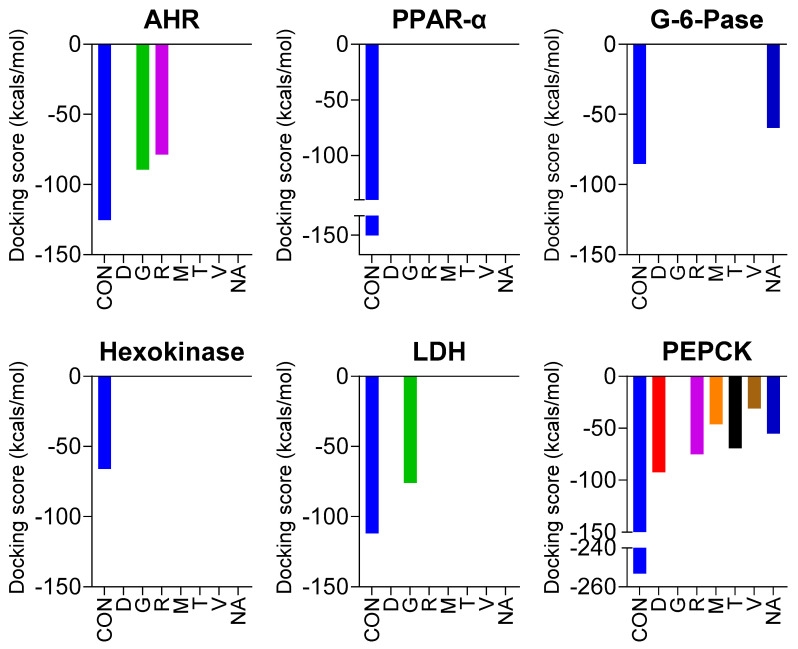
AD drugs have limited binding to AHR, PPAR-α and glycolytic enzymes. Docking score for the binding of AD drugs to AHR, PPAR-α and glycolytic enzymes is inversely proportional to the degree of binding. For abbreviations, see Table 1. Docking score (Kcal/mol) is inversely proportional to the degree of binding. Drugs are indicated as follows: D—donepezil; G—galantamine; R—rivastigmine; M—memantine; T—tropisetron; V—varenicline; NA—N-acetylcysteine. CON refers to the routine ligand for each target, listed in Supplementary Table S1.

All seven drugs docked to the kainate receptor (Figure 1), whereas none docked to the AMPA receptor (Figure 2). Only two drugs docked to the GABA_A_ receptor—donepezil (Figure 1; Table 2) and tropisetron (Figure 1; Table 6)—or the GABA_B_ receptor (tropisetron and varenicline) (Figure 1).

### 2.4. Docking to the AHR, PPAR-α and Glycolytic Enzymes

AlzD pathophysiology may involve IDO1 activation in an AHR-dependent manner that also involves impaired glycolysis. It was therefore of interest to perform molecular docking to the AHR, PPAR-α and glycolysis-related enzymes. Both glucose-6-phosphatase (G6P) and hexokinase are the gateways to glycolysis, with the former inhibiting and the latter facilitating the glycolytic process. The loss of lactate in the AlzD model described above suggests impaired lactate production either by inhibition of lactate dehydrogenase or decreased pyruvate through impaired phosphoenolpyruvate carboxykinase (PEPCK).

Docking to the AHR was observed only with galantamine (Table 3) and rivastigmine (Table 4). No drug docked to PPAR-α or hexokinase (Table 1, Figure 2). Galantamine also docked to lactate dehydrogenase (LDH), whereas N-acetylcysteine (Table 8) docked to G6P. Six of the drugs docked to PEPCK, the only exception being galantamine (Table 1 and Table 3). The docking of quinolinic acid (Figure 2) to PEPCK may indicate a possible interaction, especially in view of its well-known inhibition of this enzyme.

### 2.5. Docking to Enzymes of the Kynurenine Pathway

**Figure 3:**
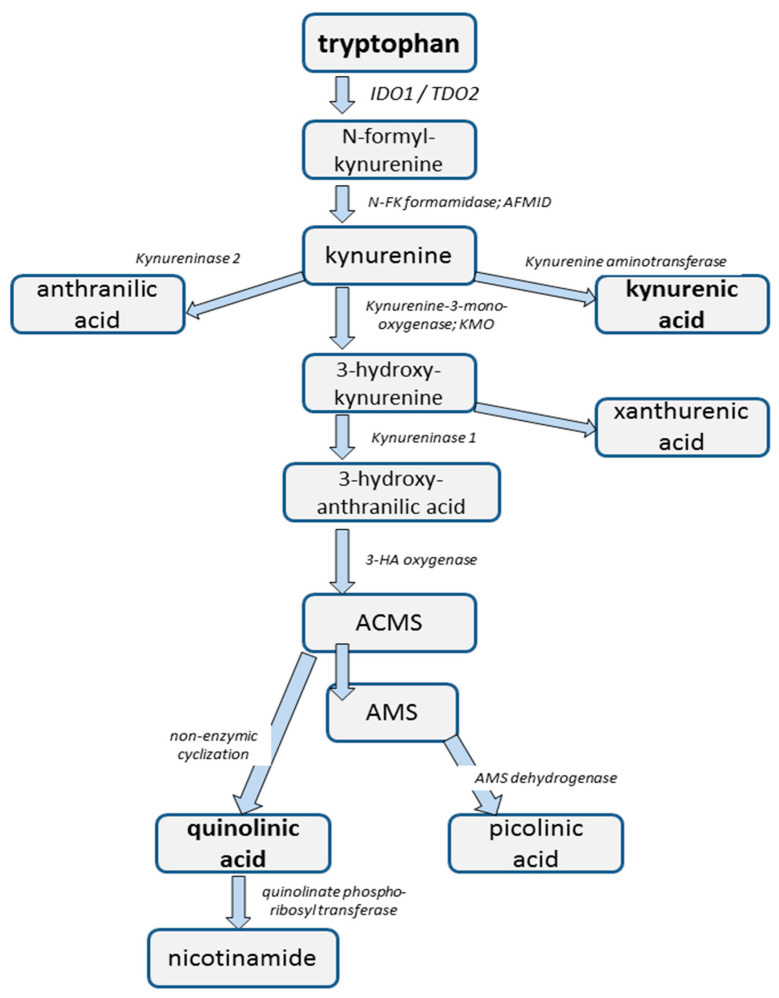
The kynurenine pathway of tryptophan metabolism. A summary of the metabolism of tryptophan along the kynurenine pathway. IDO, indoleamine 2,3-dioxygenase; TDO2, tryptophan 2,3-dioxcygenase. For other abbreviations, see Table 1.

The enzymes involved in the kynurenine pathway (Figure 3) (IDO1, TDO2, KMO, KAT-2, KYNU, 3-HAAO, ACMSD, QPRT) were selected from high-resolution holo structures where available to ensure accurate representation of the catalytic pocket. These enzymes do not undergo major conformational changes associated with activation states, so standard docking on holo-conformations is appropriate.

**Figure 4:**
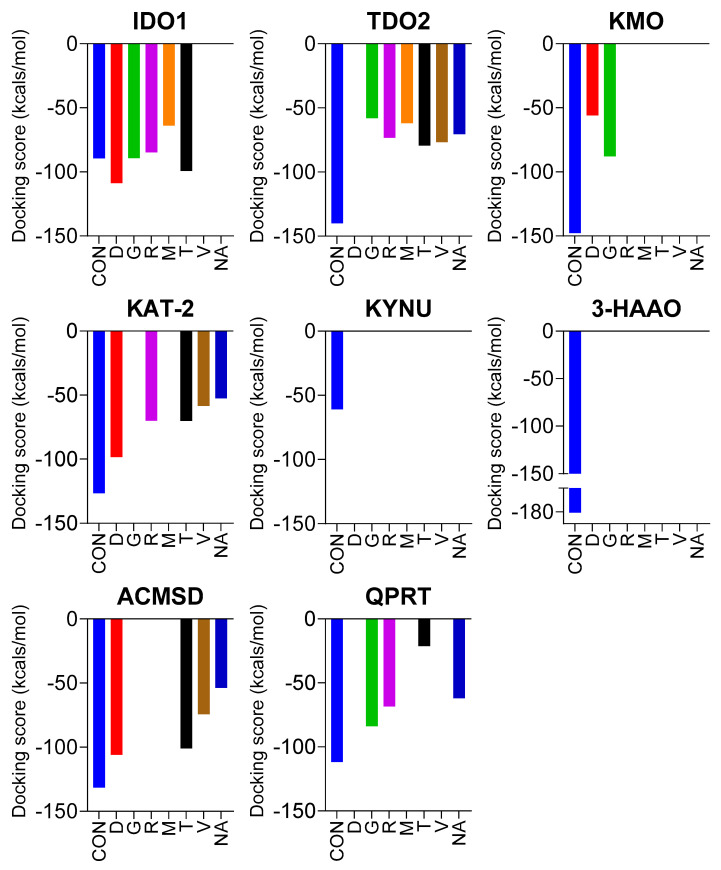
AlzD drugs dock to enzymes of the kynurenine pathway. Docking score for the binding of AD drugs to enzymes of the kynurenine pathway is inversely proportional to the degree of binding. Drugs are indicated as follows: D—donepezil; G—galantamine; R—rivastigmine; M—memantine; T—tropisetron; V—varenicline; NA—N-acetylcysteine. CON refers to the routine ligand for each target, listed in Supplementary Table S1.

The results in Figure 4 show that all of the AlzD drugs interact with some enzymes of the kynurenine pathway (summarized schematically in Figure 3), with several drugs docking to the two rate-limiting enzymes, IDO1 and TDO2. All the drugs except varenicline and NAC docked with IDO1, and all except donepezil docked with TDO2. Docking to other kynurenine pathway enzymes was variable; none docked to kynureninase or 3-hydroxyanthranilic acid 3,4-dioxygenase (3-HAAO). Donepezil docked to KMO and ACMSD, while galantamine and rivastigmine docked to QPRT. Kynurenine aminotransferase-2 (KAT-2) docked with donepezil and rivastigmine (Figure 4; Table 1).

### 2.6. Functional Assessment of IDO1 and TDO2

**Figure 5:**
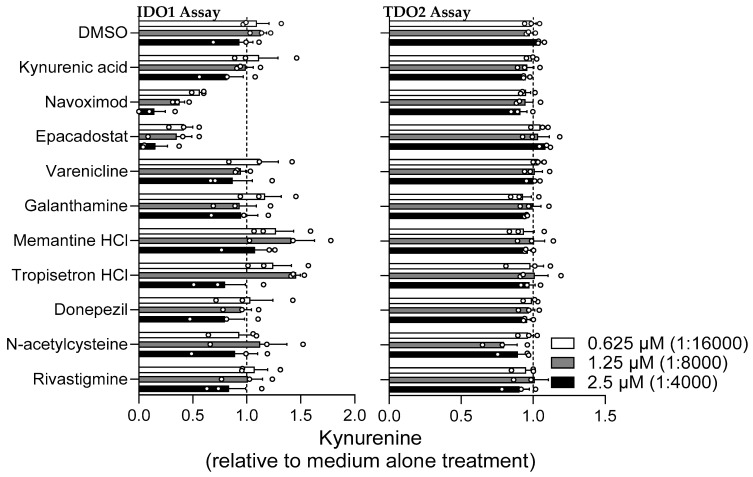
**Effects of drugs on IDO1 and TDO2 enzyme activities in HEK cells.** Relative production of kynurenine by HEK293 cells overexpressing IDO1 (**left**) or TDO2 (**right**) in the presence of kynurenic acid, IDO1 inhibitors (navoximod, epacadostat) and AD drugs at 2.5, 1.25 and 0.625 µM (equivalent to DMSO concentrations of 1:4000, 1:8000 and 1:16,000, respectively); relative to medium alone (mean ± S EM, n = 3 replicates).

As shown in Figure 5, none of the AlzD drugs exerted a significant inhibitory effect on the production of kynurenine by cultures of HEK293 cells stably transfected with IDO1 or TDO2. This contrasts with the docking ability of several of the AlzD drugs to these two enzymes (Figure 3). The implication of this mismatch is that no functional correlation can be assumed between the docking ability of a compound and its biological activity.

## 3. Discussion

The pathophysiology of AlzD involves a functional impairment of multiple neurotransmitters. Even though much attention has been paid to the roles of β-amyloid and tau protein deposits in AlzD, it remains unclear whether this is partly the result of neuroactive compound activity or whether the abnormal proteins produce changes in synaptic neurochemistry that are then responsible for specific symptoms and kinetic aspects (such as rate of development, establishment of plateaus, sensitivity and time course of treatment response). As a preliminary step towards exploring other mechanisms and as a prelude to potential future investigations, we have performed molecular docking of AlzD drugs to the crystal structures of enzymes and receptors implicated in the disorder.

The advantage of the molecular docking technique is its ability to indicate potential direct interactions between chemicals and target proteins in a pure environment free from the confounders encountered in cell culture systems in vitro or under in vivo conditions, such as absorption, metabolism, substrate concentrations, enzyme reactions, redox state, competition from other ligands, and protein binding. If docking occurs, it may provide sufficient information on the structural requirements of a ligand to guide the design and synthesis of similar and related compounds that may exhibit functional activity. If docking is not indicated for a compound with known functional activity, the approach may suggest a re-assessment of the precise target site for that activity and a re-examination of alternative target sites, some of which may have appeared functionally less interesting than the original. Combined with functional analyses, docking also provides a clue as to whether an active compound might be working via the primary ligand binding sites of the protein or is more likely to act on a distant site or even a related enzyme in a different metabolic pathway.

Nevertheless, molecular docking does have significant limitations, not least of which is its inability to provide information on the functional consequences of binding to a target site. It is for this reason that in a drug development programme, initial docking data is invariably followed by labelled ligand binding experiments in a biological system, rather than in silico. It is also the rationale for the assessment of IDO1 and TDO2 activity in the present study, to determine whether docking has any relationship to biological activity. Those functional assays, however, clearly indicate a lack of correspondence between the docking parameters of compounds and their biological activity. This in turn suggests that the progression of drug development should not be predicated on docking ability alone. The converse, however, may still apply: the ability of a compound to bind with a target site implies the possibility of a functional interaction, whether as an agonist, antagonist or one of the related activities such as inverse agonist. While there is no simple relationship between docking energy and function, a positive interaction might encourage more detailed functional exploration of lead compounds in their chemical synthesis and pharmacological analysis.

### 3.1. Acetylcholinesterase (AChE)

Inhibition of AChE is the main recognized action of galantamine, rivastigmine and donepezil. Donepezil docked to a site other than the recognized active site, possibly indicating that this drug may have interacted at one of several allosteric sites [51]. The remarkable difference in docking scores between donepezil and galantamine may reflect the differences in IC_50_ values for inhibition of cortical AChE by donepezil vs. galantamine (14 vs. 575 nM respectively) and of cortical acetylcholine release (30 vs. 646 nM respectively) [52,53,54]. The relatively weak inhibition of AChE by galantamine in functional studies in vitro is further suggested by its low level of inhibition compared to that of four new structurally related galantamine hybrids [55].

The strong docking of rivastigmine is interesting in view of its relatively low IC_50_ (3.81 µM) in enzyme assays and could be consistent with its non-competitive nature and high binding affinity in vitro [7]. It is also possible that rivastigmine is inactivated during AChE inhibition. Drugs not acting on AChE include memantine, which is primarily an antagonist at NMDA receptors, while varenicline is predominantly an agonist at α7-nAChRs and a partial agonist at α4,β2-receptors [56,57,58,59,60,61].

### 3.2. Nicotinic and Amino Acid Receptors

Galantamine has probably received more attention than any other as a dual-function compound inhibiting AChE and potentiating nicotinic receptor activation. However, despite a thorough search of the literature, no convincing, independent, repetition of the original reports that galantamine is a robust positive modulator at α7-nAChRs could be found [62,63,64,65]. While still recognising the mismatch between function and docking, this might be related to the failure of galantamine to dock to the α-7nAChR. On the contrary, a comprehensive attempt to repeat the report of allosteric potentiation of nicotinic receptors failed to show any such effect using several cell types and a wide range of ligand concentrations [10]. Indeed, in this latter study, concentrations of galantamine > 10 µM were found to be inhibitory.

These concerns about the activity of galantamine, however, might be consistent with the present data showing that galantamine docks strongly to NMDA receptors. As noted elsewhere, docking properties do not in themselves indicate a functional interaction; the binding to NMDA receptors is intriguing given the functional activity of galantamine at those sites [66,67,68]. Since galantamine is a weak and non-selective inhibitor of cholinesterases, other actions, possibly including NMDA receptor blockade, have previously been proposed as relevant to its overall pharmacology [4].

Strong docking to the NMDA receptors is equally pronounced with tropisetron, donepezil and to a lesser extent N-acetylcysteine. All seven compounds, however, docked to the kainate receptor, with rivastigmine exhibiting the greatest affinity, while no compound docked to the AMPA receptor. Although there is evidence for decreased AMPA and kainate receptor function in AlzD (see [69]), the functions of ionotropic glutamate receptors are complex and multifactorial, rendering the interpretation of the docking of AlzD drugs particularly difficult.

Studies of GABA function in AlzD have used cerebrospinal fluid or post-mortem brain, although the results are contradictory [70]. Impaired GABAergic synaptic transmission may be partly induced by Aβ [70,71,72]. The docking of donepezil and tropisetron to the GABA_A_ receptor may be relevant to understanding these effects, especially since those drugs can protect against Aβ-induced toxicity, Aβ formation and AlzD pathology.

### 3.3. AHR, PPAR -α and Glycolytic Enzymes

Using Aβ and tau mouse models of AlzD, Minhas et al. [73] reported IDO1 activation and suppression of glycolysis in an AHR-dependent manner, and that IDO1 inhibition restores glucose metabolism and long-term potentiation. Inhibition of the AHR can prevent the consequent changes in glucose metabolism. Only two of the AlzD drugs (galantamine and rivastigmine) docked to the AHR. However, neither drug interacted with PPAR-α nor with the glycolytic enzymes glucose-6-phosphatase and hexokinase, but N-acetylcysteine interacted with glucose-6-phosphatase. Only galantamine interacted with the final enzyme of glycolysis, lactate dehydrogenase. All AlzD drugs except galantamine interacted with PEPCK.

### 3.4. Enzymes of the Kynurenine Pathway

AlzD is associated with disturbed tryptophan metabolism [74,75,76,77,78,79,80,81,82,83,84], although some findings have been contradictory and could be explained by sample collection under non-fasting conditions [85,86]. Systematic reviews concluded that plasma tryptophan concentration is decreased [75] in AlzD and its metabolism is shifted towards the kynurenine pathway [82]. TDO2 is induced by the glucocorticoid cortisol, levels of which are raised in AlzD [87,88,89] and correlate with the AlzD severity [88]. In the hippocampus of AlzD patients and a triple transgenic AlzD mouse model, mRNA expression is enhanced for both TDO2 and IDO1, with TDO2 immunoreactivity colocalized with quinolinic acid, neurofibrillary tangles, tau and amyloid deposits in the hippocampus of AlzD [90,91].

It is noteworthy that TDO2 inhibition reduces AlzD-related cellular toxicity and behavioural deficits in animal models, and inhibits reversed recognition memory deficits [92]. However, that study showed that TDO2 inhibition did not affect spatial learning, memory or anxiety-related behaviour in their AlzD mouse model. TDO2 inhibition, however, lowers anxiety in other models, e.g., after *tdo2* gene deletion in mice [90], with a synergistic effect and TDO2 inhibition by allopurinol [93] or chronic nicotine treatment [94] in rats.

Future work might include consideration of the relationship between nitric oxide and the drugs used here. NO plays important roles in AD pathophysiology [95,96,97,98,99,100], with marked effects on tryptophan metabolism. It inhibits IDO1 by undermining its gene transcription and stability, but also controls haem availability to IDO1 and TDO2 through the GAPDH-haem chaperone [101]. A small increase in NO concentration increases the provision of haem to IDO and TDO2, whereas higher levels depress it [101]. It is notable that galantamine, which interacts with the AHR and LDH, also inhibits iNOS, thereby lowering NO levels [102].

Evidence strongly suggests IDO1 activation in AlzD, with involvement of Aβ, pro-inflammatory cytokines, and upregulation of AHR, IL-6 and STAT3 [103,104,105]. That Aβ is involved in IDO1 induction is suggested by its ability to increase levels of pro-inflammatory cytokines and to decrease those of the anti-inflammatory IL-10. AlzD is also associated with upregulation of the AHR. Together with IL-6 and STAT3, the AHR forms an autocrine loop that results in maintained IDO1 expression [106].

Docking to kynurenine pathway enzymes was variable. It is noteworthy that donepezil corrects the abnormal urinary levels of the KAT-2 products kynurenic acid and xanthurenic acid in rats with Aβ-induced AlzD. In addition to increased expression of IDO1 and TDO2, increased expression of 3-HAO, ACMSD and QPRT has been reported in transgenic AlzD mice and AlzD patients, mainly in the cerebellum and hippocampus [90]. In view of these strong relationships between the two dioxygenases and AlzD, the docking of AlzD drugs was intriguing. Nevertheless, in the functional assays of enzyme activity, we could not demonstrate IDO1 or TDO2 inhibition by AlzD drugs in stably transfected HEK cells. This dichotomy is a strong reminder that docking ability does not correlate with functional activity. In this case, the absence of enzyme inhibition might indicate that:

(1) higher concentrations may be required in vitro and in vivo;
(2) the AlzD drugs may inhibit the enzymes by preventing the conjugation of their apo forms with haem; many compounds, such as allopurinol [93] and some antidepressant drugs [106], inhibit TDO2 activity by this mechanism;
(3) AlzD drugs may inhibit the total TDO2 activity (elicited in the presence of added haem), but not the already active haem-containing holoenzyme. The two transfected apoenzymes may exist in HEK293 cells that are only partially saturated with haem, and in the absence of free available haem, AlzD drugs cannot act. Whereas a haem saturation of TDO2 (defined as a hundred-fold greater activity of the holoenzyme relative to total enzyme) of up to 50% has been documented for rat liver, a < 50% saturation is observed for TDO2 and IDO1 when expressed in different cell types [101]. In their supplementary material, the latter authors demonstrated a basal haem saturation of IDO1 and TDO2 of 18% and 33% respectively in transfected HEK cells, which increased to 99–100% by the addition of haem at 3 and 6 μM. As TDO2 or IDO1 induction is a major factor in the decrease in plasma tryptophan in AlzD, further assessment of potential inhibition of the enzymes as a function of haem availability might clarify the nature of the interaction between drugs and the enzymes. The importance of haem is further emphasized by the role of NO in controlling its availability to TDO2 and IDO [101,102].

## 4. Materials and Methods

### 4.1. Molecular Docking

Molecular docking was performed using the Molegro Virtual Docker 6.01 (MVD) [107,108,109,110], a widely used software as described previously from this laboratory [111,112]. The docking analysis was performed using semi-flexible docking, in which ligand molecules were treated as flexible to allow movement around rotatable bonds. The protein receptor was maintained in a rigid conformation throughout the simulation. This semi-flexible docking strategy is commonly employed in molecular docking studies to efficiently predict ligand–receptor interactions while maintaining computational reliability and reducing complexity. Docking pose selection was based on ranking and visual inspection of binding orientation rather than cluster-based filtering. Using the internal search algorithm settings of MVD, one docking simulation was performed for each ligand using 10 independent runs, where each run generated one predicted binding pose conformation or solution generated automatically by the software. The software subsequently ranked these conformations according to docking score, and the top-ranked pose with the lowest binding energy was selected for further interaction analysis. Since molecular docking is a computational predictive approach in which the generated poses represent alternative predicted binding conformations rather than biological replicates, standard statistical analysis is not appropriate. The best-ranked docking score is conventionally used for comparative binding analysis.

Compounds and target selection were based on pre-existing information on the pharmacology and clinical utility of drugs. Binding sites were selected based on existing literature on active-site residues. We also took into account pockets and conserved functional domains to ensure the docking grids were biologically relevant. When possible, the docking parameters were checked against known ligand-binding regions to improve the reliability of our results. A re-ranking procedure was employed to improve accuracy. When re-ranked, 10 independent docking runs resulted in 10 solutions. The structures of the compounds tested were imported into the MVD workspace in SDF format. All H atoms were added, and bond orders and valence states were verified prior to docking. The crystal structures of a number of human enzymes and receptors were obtained from the Protein Data Bank (https://www.rcsb.org/) and imported into the MVD work region at a resolution level of ≤2.8 Å.

Docking grid boxes were defined based on the coordinates of co-crystallized ligands (where available) retrieved from the Protein Data Bank, ensuring that the active site was accurately targeted. In cases where co-crystallized ligands were not present, grid boxes were defined using literature-reported catalytic residues, conserved active-site motifs, and previously validated binding pocket information from published studies on the same targets. This approach ensures that docking simulations were anchored to experimentally or functionally validated binding regions rather than arbitrary surface scanning. The grid dimensions were optimized to fully encompass the active-site cavity while minimizing non-specific sampling of irrelevant surface regions.

Structures of the tested compounds were prepared in UCSF Chimera, energy-minimized, and imported into the MVD workspace in Structure Data File (SDF) format. Only high-resolution crystal structures (≤2.8 Å) were selected to ensure reliable atomic positioning and accurate binding site geometry. Protein preparation was carried out using SWISS PDB Viewer (https://spdbv.unil.ch/, accessed on 14 May 2026). Missing hydrogen atoms were added, protonation states of ionizable residues (Asp, Glu, His, Lys, and Arg) were adjusted to physiological pH (7.4), and the orientations of hydroxyl, thiol, and amino groups were optimized to maximize hydrogen bonding interactions. Valences of atoms within the binding site were checked to avoid unrealistic bond lengths or angles, and the coordination states of any metal ions or cofactors were verified. The prepared protein structures were further subjected to restrained energy minimization to resolve steric clashes or unfavourable interactions introduced during the preparation process. Minimization was performed using standard force fields such as AMBER (https://ambermd.org/), CHARMM (https://academiccharmm.org/), or GROMACS (https://www.gromacs.org/), depending on the system requirements.

Docking results were evaluated using the MolDock scoring function, which produces negative scores, with lower (more negative) values reflecting stronger binding affinities. Positive scores generally indicate poor binding, often due to steric hindrance, repulsive electrostatics, or unfavourable conformational fit of the ligand within the active site. Further details of the docking are available on the Molegro software website (http://molexus.io/molegro-virtual-docker/ (accessed on 14 May 2026)).

Although assigning hydrogen atoms and adjusting protonation states to pH 7.4 were made, separate enumeration of alternative tautomers, protomers, or microspecies was not performed. Instead, docking was conducted using the dominant protonation form generated by the software under these conditions. Ligand flexibility, including ring systems, was handled during docking through the conformational search algorithm implemented in MVD, allowing exploration of energetically feasible geometries within the binding site. This strategy has been commonly adopted in docking-based studies where evaluation of relative binding trends rather than absolute free energies is the primary objective.

Our study was an exploratory screening of many protein targets that are very different from each other, and some of the targets do not have high-resolution co-crystallized ligand complexes. Others do not have experimentally validated reference inhibitors. This made it impossible to perform uniform re-docking and RMSD validation for all targets.

### 4.2. Enzymes and Receptors

MVD was used for docking and ligand map interaction along with experimental enzyme assays that support the docking trends. Rescoring/MD/MM-GBSA were not performed for all complexes. To confirm if a ligand docks to the active site of its target protein, it must bind to one or more amino acid residues at or near the active site. These can be recognized by comparing the observed residues at or near the ligand binding site with those observed with the protein’s own natural ligand (Supplementary Table S1). Accordingly, only genuine dockings at active sites are presented here.

The sources and PDB ID numbers of enzymes and receptors are listed in Table 9. The ligands were generated by assigning the missing bonds, hydrogen(s) and charges using UCSF Chimera 1.5 software. The scoring functions of the MVD and other docking details and their application have been described in our earlier work [7,55]. However, several proteins in Table 9 (GluN2B, GABRB3, GABAB, kainate receptor subunit GRIK2, AMPA receptor subunit GRIA2, and α7-nicotinic receptor CHRNA7) are components of multi-subunit, state-dependent ligand-gated ion channels. GluN2B (3JPW) represents the ligand-binding domain in complex with the antagonist ifenprodil; this inhibited closed-cleft conformation is commonly used for antagonist docking. The structures of GRIK2 (8FWR) and GRIA2 (5VHZ) were chosen because they include the ligand-binding domains that define glutamate recognition and are used in receptor–ligand simulation studies. Although they are all known to bind their endogenous ligands, there may be differences between these and other subunits which merit examination. For these receptors, PDB structures were selected based on: (a) availability of complete extracellular domains or ligand-binding domains; (b) highest resolution structures; (c) previously validated use in docking studies.

GABRB3 (4COF) is a human β3-homopentamer solved at high resolution. Although it does not represent the typical αβγ stoichiometry, it is a well-established structural model for docking because it provides a stable extracellular and transmembrane architecture used in multiple published computational studies. The GABA(B) receptor (4F11) is solved as the heterodimer Venus flytrap domain, which is the physiologically relevant ligand-binding region and suitable for orthosteric docking. For CHRNA7 (7KOX), the extracellular ligand-binding domain structure was used because it provides the orthosteric binding pocket for nicotinic agonists and antagonists. The selection of these specific conformations was based on their stability, completeness, and validated use in prior docking literature, making them appropriate for computational ligand–receptor interaction studies despite the absence of full-length structures for some receptors.

### 4.3. Enzyme Activity

In view of the potential importance of IDO1 described above and of the kynurenine pathway in general in AlzD pathology, IDO1 and TDO2 activities were also measured in the absence and presence of various concentrations of AlzD drugs. HEK293 cells were stably transfected with vectors to express human IDO1 or human TDO2. The cells were aliquoted into 96-well flat-bottom plates (100,000 cells in 125 µL of 10% foetal bovine serum, 1% P/S Dulbecco’s Modified Eagle Medium, DMEM). To each well was added 125 µL of each compound at a final concentration of 2.5, 1.25 or 0.625 µM; the 2.5 µM concentration had a DMSO (dimethylsulphoxide) concentration of 1/4000. Each plate contained a vehicle (DMSO) control and a medium-only control in duplicate. The cells were cultured overnight (20 h). The plates were centrifuged at 1500 rpm (~500 *g*) to deposit any non-adherent cells to the surface. Three aliquots of 70 µL were removed and placed in 96-well V-bottom plates for the measurement of kynurenine, and an MTT (3-(4,5-dimethylthiazol-2-yl)-2,5-diphenyltetrazolium bromide) assay was performed on cells remaining in the plate. The assay confirmed nominal differences between groups, showing the absence of significant toxicity (Supplementary Figure S1).

Kynurenine concentration was measured as follows: To 70 µL of conditioned medium in a V-bottom plate, 35 µL of 30% trichloroacetic acid (TCA) were added. A kynurenine standard (100 μM) was prepared in complete medium, from which serial 1 in 5 dilutions and a blank were made. The plate was covered with an adhesive sheet and vortexed gently to mix, then centrifuged at 2000 RPM for 20 min. The supernatant (75 µL) was transferred to a 96-well, flat-bottom plate. To each well, 75 µL of Ehrlich reagent (*p*-dimethyl-benzaldehyde in glacial acetic acid, 20 mg/mL) was added, and absorbance was measured at 490 nm, with 620 nm being used as a reference wavelength.

The assay was performed with one biological replicate per compound concentration and two biological replicates for each vehicle control condition, consistent with the exploratory nature of this study. Metabolite quantification was conducted with three technical replicates per sample to ensure measurement reliability. A two-way ANOVA with Holm–Sidak’s multiple comparisons test was applied. This analysis identified a statistically significant effect for the positive control epacadostat under IDO1-overexpressing conditions (*p* < 0.05), but not under TDO2-overexpressing conditions, further supporting the assay’s ability to detect enzyme-specific inhibition. The study included appropriate controls: positive controls (epacadostat, navoximod), a vehicle control, and a medium-only control, ensuring proper interpretation of the results.

### 4.4. Study Limitations

A limitation of this work is that no analysis of molecular dynamics (MD) was performed to evaluate the dynamic stability of docked complexes. This was because the study was designed as a comparative molecular docking investigation involving multiple approved drugs against relevant target enzymes to assess their relative binding affinities and interaction profiles. MD simulations for all ligand–target complexes would substantially increase computational complexity and extend beyond the intended objectives of this study, which were to explore binding interactions of known drugs at the molecular level rather than to develop novel lead compounds requiring extensive dynamic validation.

## 5. Conclusions

While not necessarily reflecting biological activity, the present docking study is consistent with AChE inhibition as one possible mechanism of action for several drugs currently in use for the therapy of Alzheimer’s disease. The data on kynurenine pathway enzymes suggests the need to consider future biochemical and pharmacological research on this major route of tryptophan metabolism. Although the functional assessment of IDO1/TDO2 activity indicates an absence of inhibition, this does not parallel the docking ability, indicating the need for caution in interpreting docking data, but leaving open future work on other enzymes and sites of mechanisms of functional activity. The results also indicate that kynurenic acid, a significant factor in a range of disorders, does not bind to IDO1 or TDO2, whereas most AlzD drugs do. The role of NO in AlzD could be further explored in relation to tryptophan metabolism. It is also clear that there is no simple correlation between docking ability and biological function or activity. A much greater understanding of the mechanisms and significance of docking data is required if it is to be used for meaningful studies of molecular structure.

## Data Availability

Further details on the docking parameters and sites are available from S.D.

## Supplementary Materials

The following supporting information can be downloaded at https://www.mdpi.com/article/10.3390/ijms27156555/s1.

## Abbreviations

AChE, acetylcholinesterase; α-7-nAChR, α-7-nicotinic acetylcholine receptor; ACMS, 2-amino-3-carboxymuconate-6-semialdehyde; AHR, aryl hydrocarbon receptor; GPR35, G-protein-coupled receptor-35; HBA, HBD, HBE, hydrogen bond acceptor, donor, energy; 3-HAA, 3-hydroxy-anthranilic acid; 3-HK, 3-hydroxykynurenine; IDO1, indoleamine 2,3-dioxygenase-1; Kyn, kynurenine; KMO, kynurenine monooxygenase; NAD^+^, oxidized nicotinamide–adenine dinucleotide; NMDA, N-methyl-D-aspartate; PEPCK, phosphoenol-pyruvate carboxykinase; QA, quinolinic acid; TDO2, tryptophan 2,3-dioxygenase.
