# Supplementary material for: Docking Analysis of Drugs Used in the Treatment of Alzheimer’s Disease, Using Cell Membrane Receptors and Enzymes of the Kynurenine Pathway: A Pilot Study
Source: Int J Mol Sci. 2026 Jul 23;27(15):6555. doi: 10.3390/ijms27156555 (PMC13465198; doi:10.3390/ijms27156555)
Supplement: Supplementary file 1 [file ijms-27-06555-s001.zip › Supplementary Figure S1.pdf]

Supplementary Figure S1

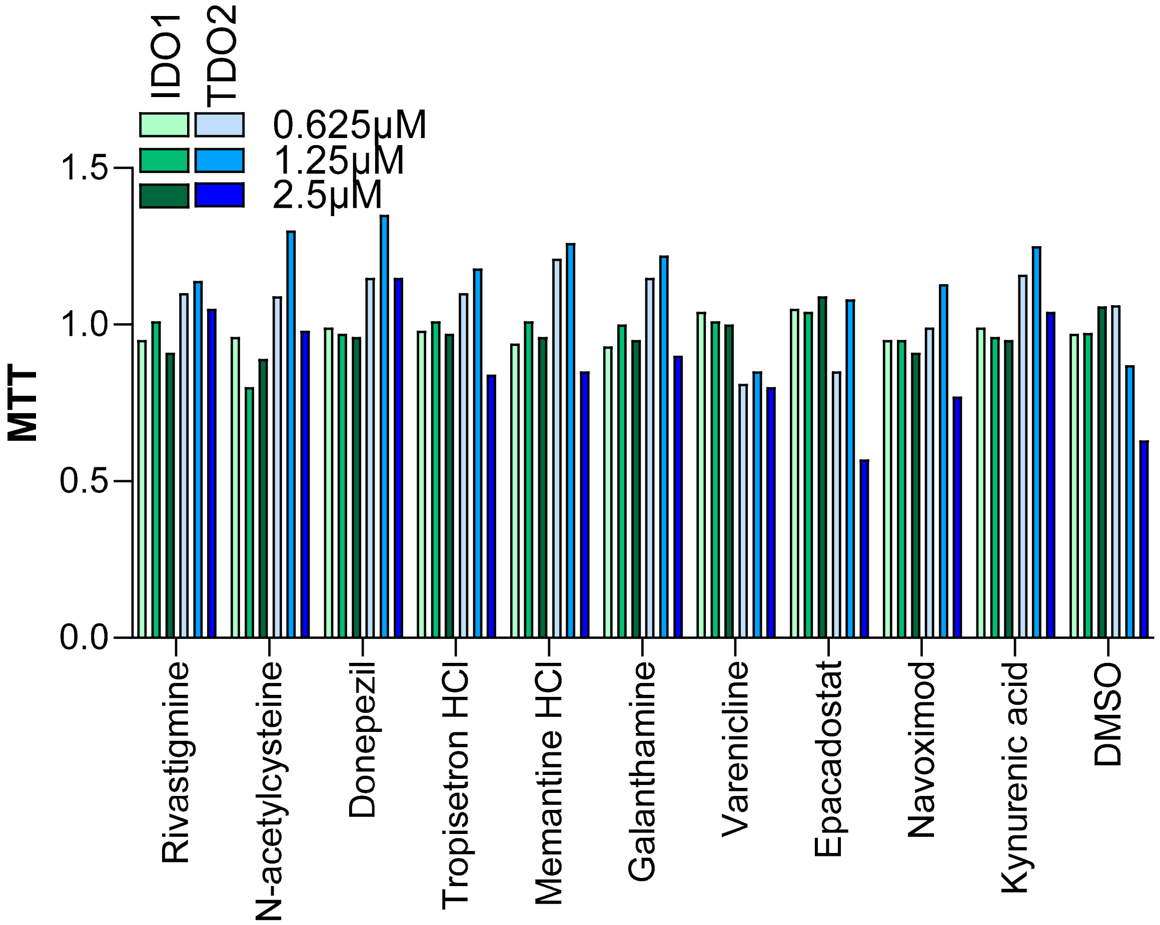

Supplementary Figure S1

Effects of drugs on MTT activity in IDO1 and TDO2 expressing HEK cells

Relative MTT activity in IDO1-HEK293 or TDO2-HEK293 cells in the presence of kynurenic acid, IDO1 inhibitors (navoximod, epacadostat) and AD drugs at 2.5, 1.25 and 0.625 µM (equivalent to DMSO concentration of 1:4,000, 1:8,000 and 1:16,000, respectively); values are relative to medium alone.
