# Supplementary material for: Docking Analysis of Drugs Used in the Treatment of Alzheimer’s Disease, Using Cell Membrane Receptors and Enzymes of the Kynurenine Pathway: A Pilot Study
Source: Int J Mol Sci. 2026 Jul 23;27(15):6555. doi: 10.3390/ijms27156555 (PMC13465198; doi:10.3390/ijms27156555)
Supplement: Supplementary file 1 [file ijms-27-06555-s001.zip › Supplementary Table S1.pdf]

Supplementary Table S1

Target	Control ligand	Score
**Figure 1**		
AChE	Dihydrotanshinone I	-125.4
α-7-ACh	Indoxyl acetate	-39.3
NMDA	NAAG	-95.1
Kainate	Glutamate	-91.4
AMPA	Quisqualate	-103.4
$GABA_A$	Benzodiazepine	-62.2
$GABA_B$	γ-aminobutyric acid	-67.9
**Figure 2**		
AHR	CYS-LEU-VAL-ALA-ILE-GLY-ARG-LEU	-125.3
PPAR-α	HW3/APHM6	-150.1
G-6-Pase	H4B Tetrahydrobiopterin.	-85.3
Hexokinase	604 (PMID: 38529190)	-66.0
LDH	Fructose 1,6-bisphosphate (FBP)	-111.8
PEPCK	Phosphoenolpyruvate carboxykinase (ATP)	-253.2
**Figure 3**		
TDO2	Tryptophan	-139.5
IDO1	N-hydroxyamine	-89.5
KMO	7ZR (Ro 61-8048)	-147.6
AADAT	Pyridoxal 5'-phosphate	-126.4
KYNU	Cysteine S-oxide	-61.0
3-HAAO	ARG-HIS-LYS-FDL	-180.7
ACMSD	TES-1025	-131.6
QPRT	Phthalic acid	-111.8

Table S1. Molecular docking of the target's own control ligand target drugs to the crystal structures of kynurenine pathway enzymes. The data are re-ranked docking scores (KCal / mol)
